# Tracking of [^14^C]Polystyrene Nanoplastics in Pregnant Mice

**DOI:** 10.1002/advs.202523995

**Published:** 2026-03-24

**Authors:** Olga Khaybullina, Pallavi Sarkhel, Outi M. Keinänen

**Affiliations:** ^1^ Department of Chemistry University of Alabama at Birmingham Birmingham Alabama USA; ^2^ Department of Radiology University of Alabama at Birmingham Birmingham Alabama USA

**Keywords:** biodistribution, carbon‐14, maternal–fetal transfer, nanoplastics, polystyrene nanoparticles, pregnancy

## Abstract

Micro‐ and nanoplastics (MNP) are increasingly reported in the reproductive and maternal organs and tissues, yet their translocation and accumulation during pregnancy remain poorly quantified – often the material used as MNP is heavily modified. Herein, we have used unmodified polystyrene (PS) nanoparticles of three sizes (33 ± 11, 246 ± 81, and 1052 ± 189 nm) with covalent ^14^C backbone labels to track biodistribution in late‐gestation mice after intranasal (IN) or intravenous (IV) administration. Tissue collection after administrations revealed exposure route‐dependent nanoplastic accumulation. After IN exposure, the majority of the nanoplastics were retained in the lungs or excreted through the GI tract, whereas IV administration exhibited size‐dependent accumulation in the liver and spleen, and a lung uptake consistent with microvascular interactions. We did not detect PS in placentae or fetuses after IN administration. Placentae exhibited low concentrations of PS (0.10%–0.15% of the injected dose) after IV administration, indicating restricted transplacental transfer even after direct intravenous exposure to relatively large amounts of nanoplastics. These findings indicate that exposure route and particle size principally govern early disposition during pregnancy. Importantly, the covalently incorporated radiolabel (^14^C) allows quantitative tracking of unmodified pure polystyrene particles and preserves the material's intrinsic behavior.

## Graphical abstract

This study investigates [^14^C]polystyrene nanoplastic ([^14^C]PS) translocation in late‐stage pregnant mice after intranasal (0.5 mg of [^14^C]PS on GD12, GD14, and GD16, *n* = 6) and intravenous administration (1.5 mg of [^14^C]PS on GD16, *n* = 6). ^14^C‐radiolabel allows quantitative tracking of unmodified polystyrene nanoplastics. Polystyrene nanoplastics have limited maternal‐fetal translocation after intranasal and intravenous administration.

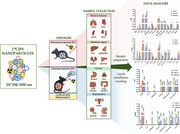

## 1 Introduction

Plastics are an irreplaceable part of our daily routine, and due to their chemical inertness, are mostly non‐biodegradable and stay in the environment for long periods of time. The total amount of plastic generated is significant: global production reached 413.8 million tons in 2023 (up from 400.4 million tons in 2022) [1]. Significant contaminants include tire wear, approximated at around 5–6 million tons annually, and the laundering of textiles [2, 3]. Over time, large plastic waste in the environment undergoes fragmentation and degradation into smaller and smaller pieces, finally ending up as tiny particles called nanoplastics [4]. Nanoplastics have been gaining increasing attention concerning their potential impacts on human health and ecological systems [5, 6, 7]. According to accepted criteria in environmental toxicology, nanoparticles (NPs) are defined as entities smaller than 1 micrometer [8, 9, 10, 11]. NPs can not only play a role as pollutants but also have the potential to act as carriers for plastic additives and adsorbed contaminants, such as heavy metals and PFAS [12, 13]. Furthermore, they may also release monomers during the weathering process, posing additional environmental hazards [5, 7, 14]. These concerns have called for comprehensive monitoring and precise determination of nanoplastic accumulation and contamination in the environment, consumer products, and living systems [10, 13, 14]. Since nanoplastics have been detected in various environmental compartments and consumer products, it is not surprising that they have been detected in human samples as well [15, 16]. Moreover, NPs have even been found in the human placenta, indicating maternal‐fetal exposure and raising concerns about a potential threat to the maternal reproductive system and fetus development [17, 18, 19].

Microplastics have also been reported in human neonatal meconium, which is consistent with the plausibility of prenatal exposure; however, meconium is an indirect endpoint and does not by itself establish transplacental transfer of intact particles. Reported findings vary across studies and can depend on analytical approach, the size fraction interrogated, and contamination control procedures. For example, microplastics have been detected in placenta and meconium in clinical and mother–infant paired studies [20, 21], while other work has reported non‐detection in meconium under their analytical workflow and detection limits [22], underscoring that outcomes can be method‐ and sensitivity‐dependent. In addition, because microplastics are ubiquitous in indoor air and laboratory environments, low‐biomass biological matrices are vulnerable to background contamination introduced during sampling and processing (e.g., airborne deposition and polymer‐containing consumables), making procedural blanks and stringent QA/QC essential for interpretation [23]. Several experimental studies further support concern for developmental susceptibility by reporting neurodevelopmental outcomes after maternal or early‐life exposure to polystyrene micro‐/nanoplastics. For example, Jeong et al. reported that maternal PS nanoplastic exposure was associated with altered neurodevelopmental endpoints in offspring, including brain abnormalities and functional deficits [24]. Similarly, Yang et al. reported fetal thalamic injury following gestational coexposure to polystyrene micro‐ and nanoparticles, consistent with oxidative‐stress‐linked apoptosis in the developing brain [25]. In addition, Zou et al. showed that neonatal exposure to PS nanoplastics can disrupt microglia‐mediated synaptic pruning and lead to persistent social behavioral deficits in adulthood [26]. Collectively, these studies emphasize that even during developmentally sensitive windows, PS micro‐/nanoplastic exposures can be associated with measurable neurodevelopmental effects, motivating quantitative approaches that directly constrain particle biodistribution and the magnitude of fetal exposure across routes and particle sizes.

Humans may encounter NPs via various pathways, including ingestion, inhalation, and, to a lesser extent, dermal absorption. The topic of ingestion exposure is currently under active investigation since NPs have been detected in all investigated food and drink products [7, 14, 27]. For example, a study on single‐particle chemical imaging conducted in 2024 estimated the presence of approximately (2.4 ± 1.3) × 10^5^ plastic particles per liter in regular bottled waters, about 90% of which were nanoplastics [27]. Inhalation is another primary route for nanoplastic exposure. Recent studies have reported NP concentrations of approximately 200–600 nanograms per cubic meter in urban air [28] and around 20–110 nanograms per cubic meter at remote alpine sites [29], indicating a persistent airborne burden – including indoors, where levels are often higher. In comparison to ingestion and inhalation, dermal uptake appears limited for intact skin. Human‐skin studies with polystyrene (PS) NPs mostly show retention in the stratum corneum and hair follicles, with noticeable penetration only when the barrier is damaged [6, 30].

Against this exposure backdrop, pregnancy warrants particular caution; however, whether nanoplastics can traverse the placental barrier and reach the fetus remains uncertain. Nevertheless, studies in animal models using PS NPs have reported placental perturbations, fetal growth restriction, and, in some settings, translocation from the maternal to the fetal circulation; however, interpretation remains uncertain because common tracking methods can bias biodistribution (e.g., dye leaching, probe‐induced surface changes, tissue autofluorescence), raising the possibility of artifactual “transfer” [31, 32].

A key challenge lies in the ability to track NPs in complex matrices, keeping their intrinsic properties and surface chemistry. As previously mentioned, fluorescent labels may leak or alter the biodistribution of the particles and cause aggregation due to their bulky hydrophobic nature. Additionally, tissue autofluorescence may be incorrectly interpreted as signal, potentially resulting in superficial “translocation” [31, 32]. Magnetic approaches, such as Fe_3_O_4_‐doped PS for magnetic resonance imaging, can effectively facilitate detection and separation; however, they may alter intrinsic properties, such as density or magnetization, encourage aggregation, and typically require substantial iron loadings, which may influence corona formation and clearance pathways [33]. Radiolabeling with ^64^Cu or ^89^Zr for real‐time tracking with positron emission tomography (PET) provides high sensitivity and quantitative whole‐body imaging, while requiring only minimal surface modification of the nanoplastic particles [34, 35, 36, 37]. However, PET labels require strict control over the degree of modification and precise control of label stability to avoid any leaching that can interfere with the actual biodistribution. In addition, positron emitters, such as ^64^Cu (t_1/2_ = 12.7 h) and ^89^Zr (t_1/2_ = 3.3 d), tend to have rather short half‐lives considering long‐term or chronic exposure studies.

To minimize any possible alterations, we synthesized radiolabeled PS nanoparticles by covalently incorporating ^14^C (t_1/2_ = 5700 y) into the polymer backbone of PS. This radiolabeling technique preserves the intrinsic surface characteristics of the particles and allows the quantitative determination of PS NP biodistribution through liquid scintillation counting (LSC). ^14^C‐radiolabeling has become a valuable technique in understanding the in vivo behavior of a variety of compounds and could offer unique advantages to the study of micro‐ and nanoplastics and other environmental pollutants. First, the detection of radioactivity is quantitative and linear. Second, tracking even in very low concentrations is possible due to the unparalleled sensitivity of radioactivity measurement. Third, there is no ^14^C background signal that would hamper the interpretation of the results. Finally, since most compounds contain carbon, ^14^C‐labeling enables the creation of an exact radiolabeled replicate of the compound under investigation.

Here, we have investigated the ability of ^14^C‐labeled PS NPs to cross the placenta into developing fetuses using two different administration routes: intranasal and intravenous. Intranasal administration was chosen to recapitulate the real‐world exposure humans are subjected to. In a recent report, micro‐ and nanoplastics were found in human blood [16] To have clarity on where the particles in the blood flow locate during pregnancy, we administered PS NPs intravenously to pregnant mice. This study is the first to use unmodified pristine polystyrene nanoplastic particles to investigate their accumulation in a pregnant mammalian model. We hope that our investigation shows the potential of ^14^C‐labeling in the quantification of environmental pollutant accumulation. Here, we used ^14^C‐labeled polystyrene particles, but ^14^C‐labeling can be extended to other polymer materials as well. Moreover, ^14^C‐labeled materials could be used in chronic exposure investigations that are required to fully recapitulate real‐world human exposure.

## 2 Results and Discussion

### 2.1 Nanoparticle Characterization

**FIGURE 1:**
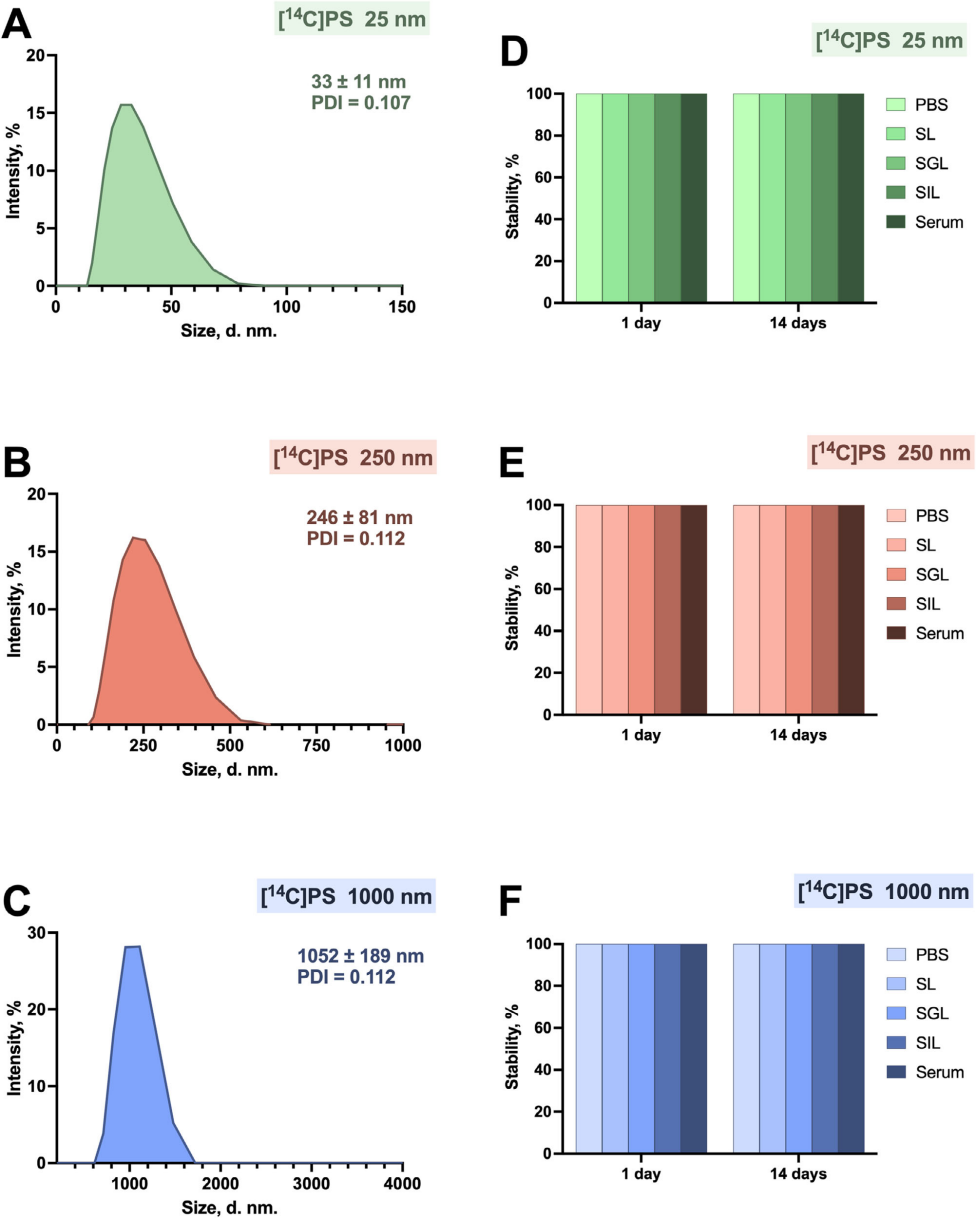
Polystyrene nanoparticle (PS NPs) characterization. (A–C) Hydrodynamic size distributions measured by dynamic light scattering (DLS) in water at 25°C for nominal 25, 250, and 1000 nm [^14^C]PS (mean ± SD, PDI shown). (D–F) Radiochemical/chemical stability at 37°C for day 1 and 14 in PBS, SL, SGL, SIL, and mouse serum, assessed by iTLC‐SG (n‐hexane/ethyl acetate 95:5, v/v) and quantified by LSC. Across all media, ^14^C recovery remained 100%, and styrene monomer was not detected (below LOD). Panels D, E, and F correspond to the 25, 250, and 1000 nm cohorts, respectively.

DLS confirmed intensity‐weighted diameters (mean ± SD) of 33 ± 11 nm (PDI = 0.107), 246 ± 81 nm (PDI = 0.112), and 1052 ± 189 nm (PDI = 0.112) for the nominal 25, 250, and 1000 nm NPs, respectively (Figure 1A–C). After 14 days at 37°C in PBS, SL, SGL, SIL, and mouse serum, samples retained 100% of the initial ^14^C signal, and styrene monomer was not detected (below LOD) (Figure 1D–F).

Electrophoretic light scattering (Zetasizer Nano ZS, 25°C, disposable folded capillary cell, 173° backscatter, Smoluchowski model) in deionized water yielded ζ‐potentials of −35 ± 9 mV (25 nm), −30 ± 6 mV (250 nm), and −40 ± 6 mV (1000 nm). These magnitudes indicate electrostatically stable dispersions under low ionic strength, and the modest differences across sizes fall within uncertainty rather than reflecting a true size effect.

### 2.2 Intranasal Exposure

**FIGURE 2:**
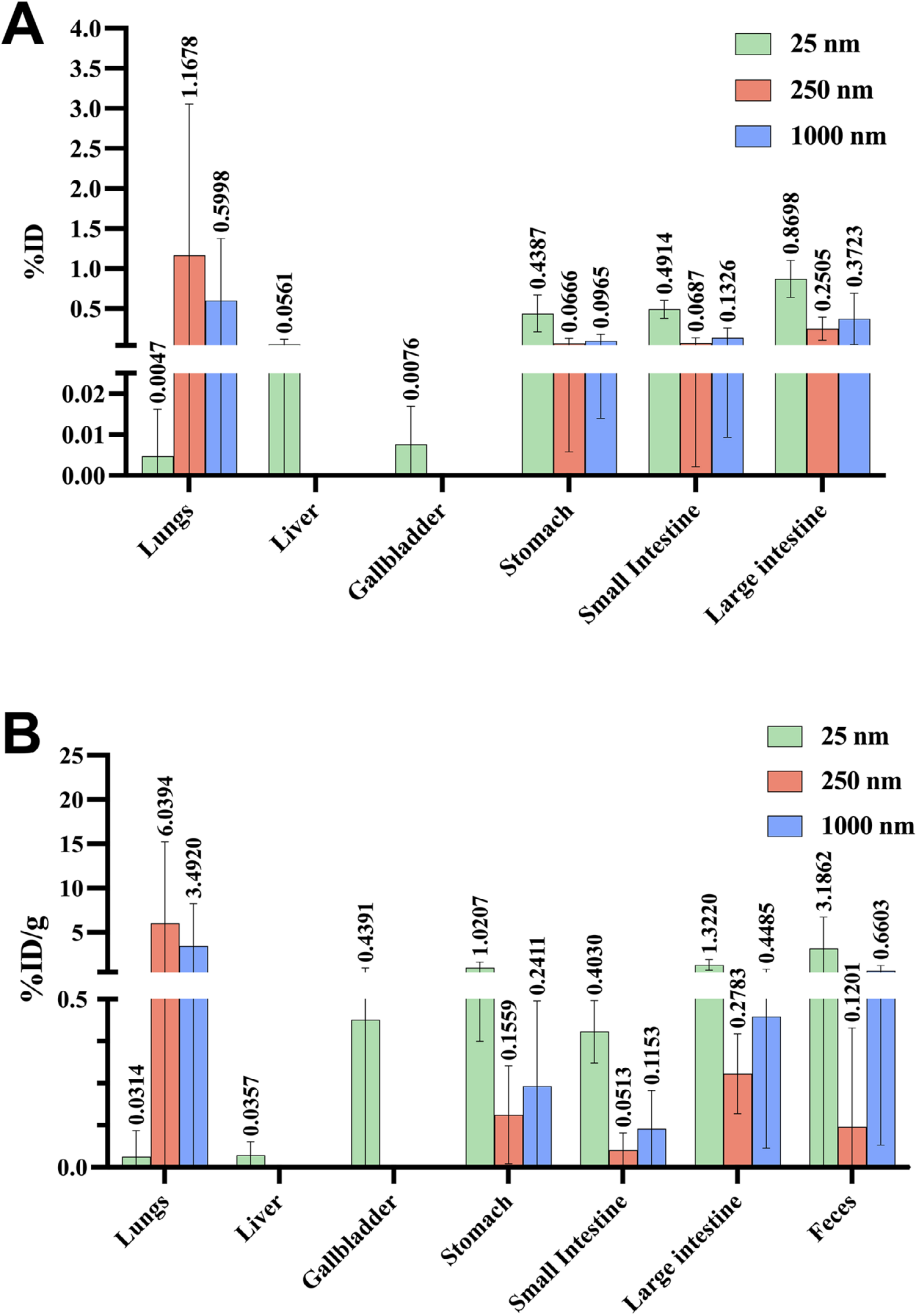
Biodistribution of [^14^C]polystyrene nanoparticles (PS NPs) in late‐gestation mice after intranasal (IN) administration. (A) Biodistribution as percentage of injected dose (%ID). (B) Organ mass‐normalized biodistribution (%ID/g). Mice received three intranasal doses on GD12, 14, and 16 (50 nCi per dose, 25, 250, or 1000 nm, *n* = 6), necropsy GD17 (24 h after the last administration). Bars show mean ± SD. Organs shown: lungs, liver, gallbladder, stomach, small intestine, large intestine, and feces (GI segments include luminal contents).

After intranasal administration, lung‐associated signal did not increase monotonically with particle size, pairwise comparisons were not statistically significant after Holm–Šídák correction (25 vs 250, *p =* 0.2, 250 vs 1000, *p =* 0.5, 25 vs 1000, *p =* 0.1, %ID: Figure 2A, %ID/g: Figure 2B). With the smallest particles (25 nm), a GI‐dominant profile with the highest ^14^C signals in the stomach, small/large intestines, and feces was observed. Among the GI tissues, significantly higher radioactivity was detected in the small intestine (*p* = 0.003) and large intestine (*p* = 0.01) (%ID, Figure 2A) for 25 nm particles when compared to 250 nm particles. This pattern is consistent with airway‐to‐GI transport via the mucociliary escalator: particles deposited on the nasal/airway epithelium are entrapped in mucus, moved toward the oropharynx by ciliary beating, and subsequently swallowed, entering the gastrointestinal tract. This mechanism provides a straightforward mechanistic explanation for the strong GI/fecal activity after intranasal exposure – particularly for 25 nm PS – while hepatobiliary uptake remained minimal and extra‐pulmonary distribution was modest at 24 h.^[^ ^35^ ^]^ This pattern was consistent after mass normalization: 25 nm exceeded 250 nm in the small intestine (*p* = 0.00005) and large intestine (*p* = 0.007), and also exceeded 1000 nm in the small intestine (*p* = 0.0008) and large intestine (*p* = 0.02) (%ID/g, Figure 2B). In contrast to previous reports of placental/fetal translocation in other NP exposure models [38, 39], ^14^C signals were not detected in the placenta or fetus. No above‐background ^14^C signal was detected in maternal or fetal brain at 24 h post‐administration for either exposure route, indicating that any brain‐associated burden was below the sensitivity of LSC under the conditions tested. Prior reports have described CNS‐related outcomes following developmental exposure to polystyrene micro/nanoplastics, where brain involvement is inferred from neuroinflammation/oxidative stress, histopathology, or behavioral endpoints. Differences across studies likely reflect exposure route and dosing paradigm, particle size/surface chemistry, gestational timing, post‐exposure timepoint, and methodological differences in detection and quantification. Collectively, our data provide quantitative bounds on brain‐associated signal at 24 h for [^14^C]PS nanoparticles across sizes and routes in late gestation. This signifies that translocation is sensitive to particle chemistry, size, dose, gestational timing, and exposure route [36, 37]. [^14^C]PS NP dispersions used here consisted only of pure PS with radiolabel covalently incorporated into the backbone (no fluorophores or surface ligands), minimizing dye‐related artifacts. Observed accumulation profiles therefore reflect route‐ and size‐dependent behavior of PS rather than label artifacts [31, 32]. Overall, the data indicate a prominent GI/fecal signal for 25 nm after intranasal administration, consistent with mucociliary clearance and airway‐to‐GI transport, while the lung‐associated signal showed no monotonic size dependence, and extra‐pulmonary uptake remained limited at 24 h. This differs from the liver/spleen‐weighted profiles typical after IV delivery, where opsonization and size‐dependent phagocytosis steer particles into the reticuloendothelial system [40, 41].

### 2.3 Intravenous Exposure

**FIGURE 3:**
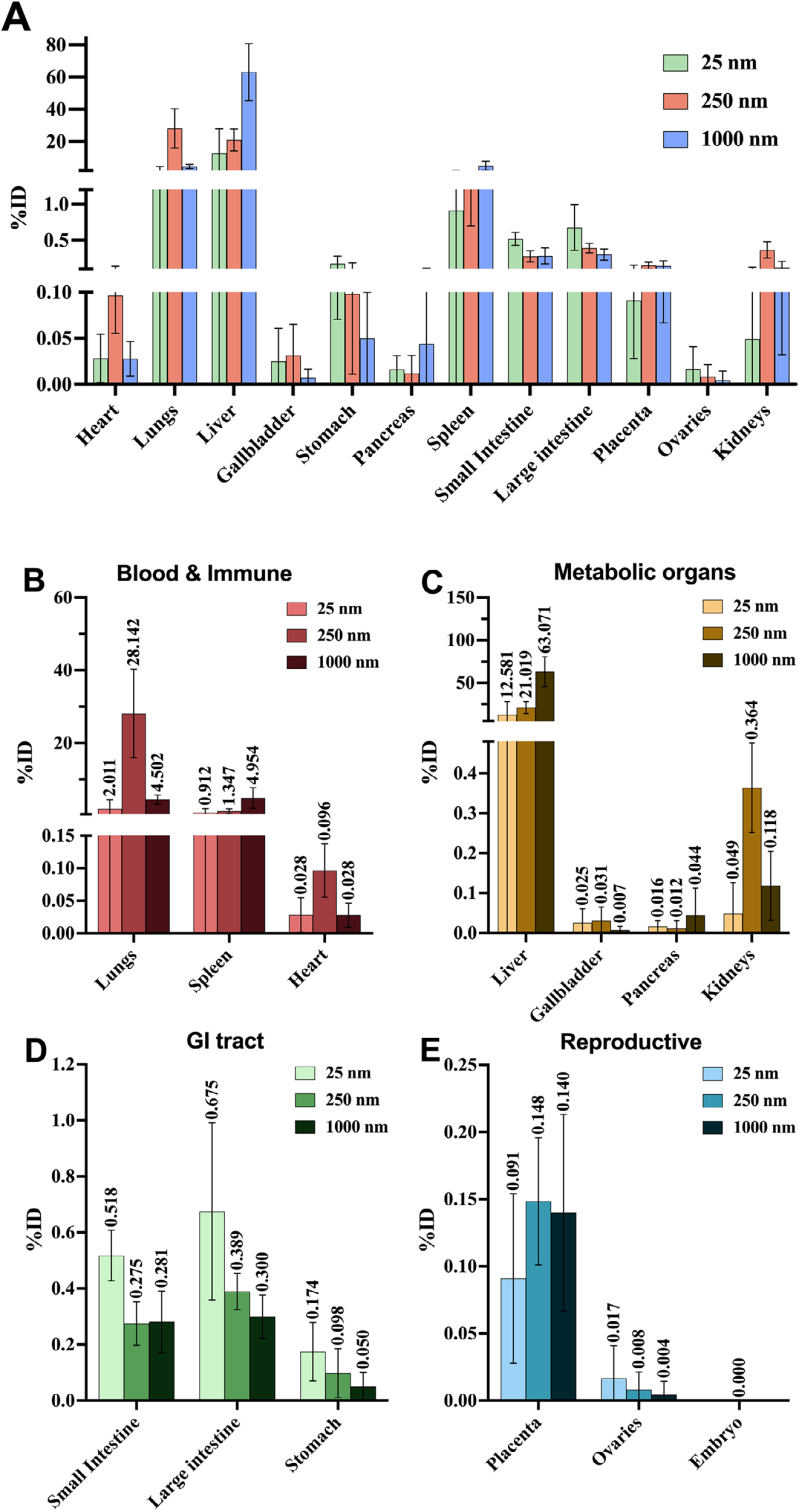
Intravenous biodistribution of [^14^C]polystyrene nanoparticles (PS NPs) in late‐gestation mice: organ burden (%ID). Dams received a single tail‐vein bolus on GD16 (150 nCi, 1.5 mg, *n* = 6 for 250 and 1000 nm, *n* = 5 for 25 nm), necropsy GD17. Bars show mean ± SD. Intestinal segments include luminal contents; fetuses/placentae were pooled per litter. Panels: (A) %ID across all organs. (B) Blood & immune: lungs, spleen, heart. (C) Metabolic organs: liver, gallbladder, pancreas, and kidneys. (D) GI tract: stomach, small intestine, large intestine. (E) Reproductive system: placenta, ovaries, fetus.

**FIGURE 4:**
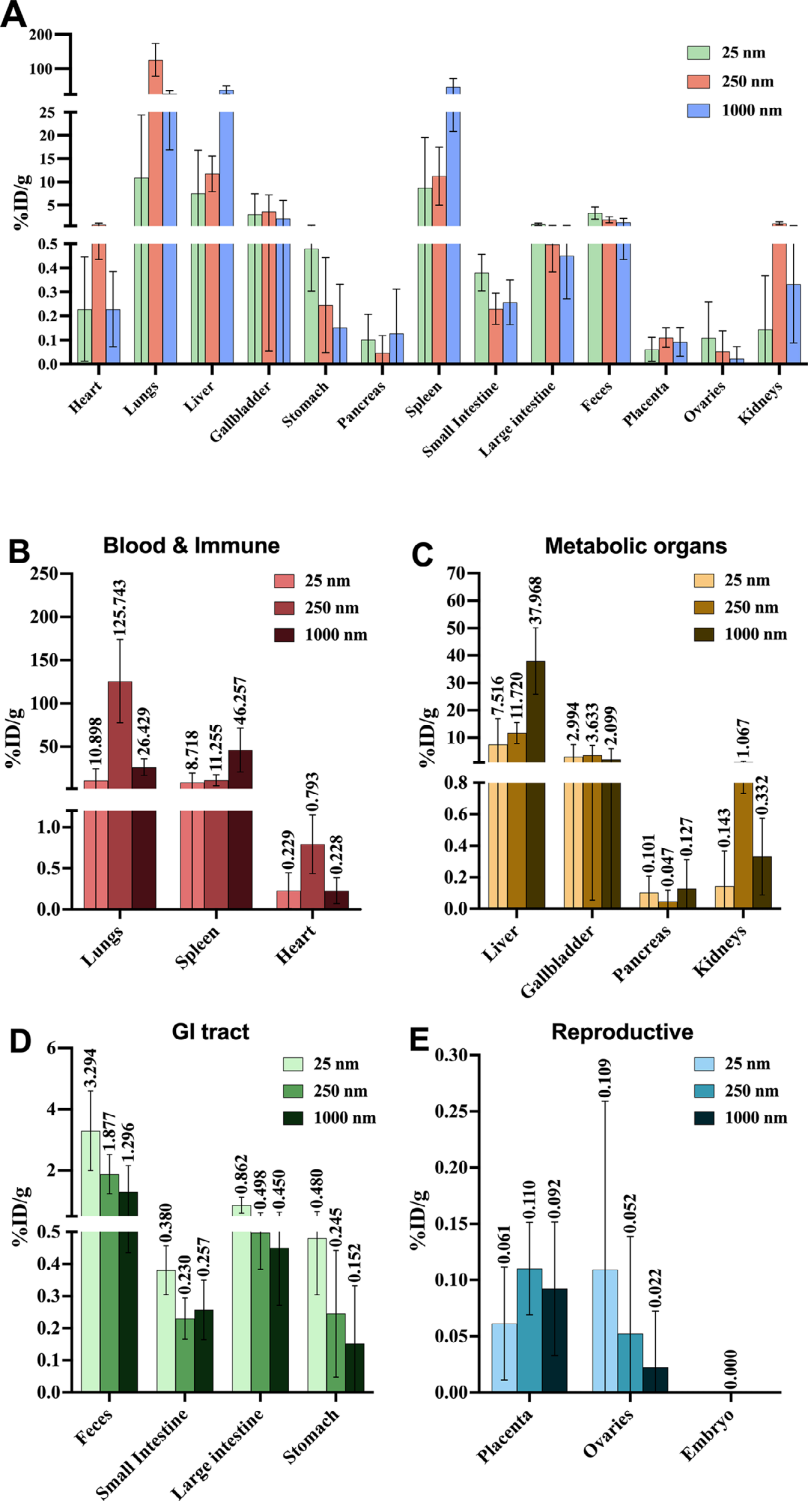
Intravenous tissue uptake of [^14^C]polystyrene nanoparticles (PS NPs) in late‐gestation mice (24 h): mass‐normalized uptake (%ID/g). Experimental details as in Figure 3. Bars show mean ± SD. Intestinal segments include luminal contents, fetuses/placentae were pooled per litter. Panels: (A) %ID/g across all organs. (B) Blood & immune: blood, lungs, spleen, heart. (C) Metabolic organs: liver, gallbladder, pancreas, and kidneys. (D) GI tract: stomach, small intestine, large intestine, feces. (E) Reproductive system: placenta, ovaries, fetus.

Intravenous administration of [^14^C]PS NPs yielded RES‐dominant biodistribution profile (%ID: Figure 3, %ID/g: Figure 4), which is consistent with typical biodistribution patterns of nanoparticles [35, 41, 42]. Larger particles displayed the highest uptake in the liver, which is consistent with opsonization and sequestration in hepatic sinusoids and macrophages. The liver predominated with a distinct size dependence (%ID: Figure 3C, %ID/g: Figure 4C). Consistent with this, hepatic activity was higher for 1000 nm compared with 25 nm (*p* = 0.001, %ID) and compared with 250 nm (*p* = 0.004, %ID), and remained significant after mass normalization (1000 vs 250, *p* = 0.007, 1000 vs 25, *p* = 0.003, %ID/g). Similarly, size‐dependent uptake was observed in the spleen (%ID: Figure 3B, %ID/g: Figure 4B), aligning with secondary reticuloendothelial capture, but size‐dependent differences did not reach statistical significance.

A notable and highest lung uptake was observed with the 250 nm particles: 28 ± 12%ID (250 nm) vs 2 ± 2%ID (25 nm) and 4 ± 1%ID (1000 nm) (25 vs 250, *p* = 0.003, 250 vs 1000, *p* = 0.005), and 126 ± 48%ID/g (250 nm) vs 11 ±13%ID/g (25 nm) and 25 ± 10%ID/g (1000 nm) (25 vs 250, *p* = 0.001, 250 vs 1000, *p* = 0.003) (Figures 3B and 4B). This pronounced 250 nm pulmonary signal is consistent with size‐dependent first‐pass retention in the pulmonary microvasculature reported for nanoparticles, whereas the 25 and 1000 nm groups showed lower lung‐associated burdens under the same conditions [43, 44]. Additional size effects were observed in the kidneys, with 250 nm differing from 25 nm (%ID: 25 vs 250, *p* = 0.0004, %ID/g: 25 vs 250, *p* = 0.0005) and from 1000 nm after mass normalization ((%ID: 250 vs 1000, *p* = 0.003, %ID/g: 250 vs 1000, *p* = 0.002) (Figures 3B and 4B). The radioactivity concentrations in the blood were close to background levels (Figure 4B), suggesting rapid tissue association. The formation of a protein corona is possibly influencing the route‐dependent biodistribution observed in this study. Nanoparticles exhibit a rapid adsorption of proteins in biological fluids, resulting in the formation of a dynamic corona that has the potential to modify their biological identity, influence opsonization, and affect uptake by the mononuclear phagocyte system. This phenomenon aligns with the pronounced retention in the liver and spleen observed following intravenous administration. The composition of the corona has been demonstrated to affect interactions with biological barriers, such as the placenta, in models utilizing polystyrene nanoparticles [45]. Although corona composition was not evaluated here, these considerations provide a mechanistic framework for interpreting the RES‐dominant profiles and limited extrahepatic/transplacental signals observed in this study. Uptake in the GI segments and feces was low at 24 h (Figures 3D and 4D) [46]. Radioactivity levels in the placenta and fetus remained low across all sizes (%ID: Figure 3E, %ID/g: Figure 4E), and no statistically significant size‐dependent differences were detected in the placenta (25 vs 250, *p* = 0.1, 250 vs 1000, *p* = 0.8, 25 vs 1000, *p* = 0.3, %ID). These findings are consistent with restricted placental translocation following a single systemic exposure and align with pregnancy‐focused reviews highlighting size‐, surface‐, and route‐dependent determinants of maternal–fetal transfer [47, 48, 49]. In contrast to our study, Huang et al. have reported that fluorescently‐labeled carboxylate‐PS particles (20‐500 nm) were detected in the placenta and multiple fetal organs within 4 h after IV injection at embryotic day 17 [39]. In another investigation, Kenesei et al. intravenously administered fluorescent carboxylate‐PS particles (50‐90 nm) to pregnant mice and detected signals in placental lacunae at 5 min, that was cleared by 4 days, whereas no uptake was observed in the embryos at any time point after administration [50]. The differences between the aforementioned studies and our study clearly highlight the importance of surface properties of the used particles. The RES dominance indicates intrinsic systemic disposition rather than dye leaching or coating‐driven recognition because, as mentioned above, the particles used in our investigation were pure PS with covalently attached ^14^C. In contrast to IN exposure, which was lung‐first with 25 nm showing GI‐dominant clearance, the IV route reorders the hierarchy to a liver‐first profile with a route‐specific 250 nm pulmonary first‐pass effect, demanding cross‐route comparison.

### 2.4 Cross‐Route Comparison: Intranasal vs Intravenous

The IV cohort acts as a systemic disposition benchmark instead of a dose‐equivalent comparator to the repeated IN regimen, so we will focus on rank‐ordering and route‐specific features. IN exposure was lung‐first and size‐dependent, with a 25 nm GI‐dominant signature consistent with mucociliary clearance (Figure 2A–B) [35, 51]. Intravenous exposure was RES‐first: liver predominance escalated with increasing size (13 ± 15 to 21 ± 7 to 61 ± 17%ID for sizes of 25, 250, and 1000 nm, respectively, Figure 3C, with same pattern with %ID/g in Figure 4C), while a significant lung signal was observed solely with the 250 nm particles (28 ± 12%ID and 126 ± 48%ID/g, Figures 3B and 4B), aligning with first‐pass pulmonary microvascular retention [35, 42, 52]. Comparing routes, for 250–1000 nm particles, the lung uptake was higher after IN, whereas the liver uptake was higher after IV. GI activity at 24 h was comparable between IN and IV (no main effect of route), while size significantly influenced distribution (Figures 2, 3, 4). Placenta and fetus remained low at 24 h for both routes (Figures 2, 3E, 4E), indicating limited transplacental transfer irrespective of entry route [47, 48, 49].

Since the NPs were chemically unmodified PS with a covalently incorporated ^14^C‐label, the observed contrasts demonstrate the intrinsic effects of size and exposure route. In practice, they reflect the biofluids first encountered and their clearance mechanisms (mucociliary vs reticuloendothelial), rather than dye‐ or ligand‐dependent surface artifacts.

### 2.5 Blood Analysis

In the IN groups, WBC, RBC, HCT, and PLT values of PS NP dosed mice remained comparable to PBS controls (Figure S2). These findings suggest that an IN exposure over one week did not substantially disrupt hematopoietic balance. In the serum biochemistry panel, most liver and kidney markers remained within control ranges, AST levels showed a modest, non‐significant increase in the 25 and 250 nm groups (25 nm: 235 ± 136 U/L, *p* = 0.2, 250 nm: 231 ± 128 U/L, *p* = 0.2, control: 153 ± 10 U/L), and ALT was higher but likewise non‐significantly so in the 250 nm group (83 ± 64 U/L, *p* = 0.1, control: 32 ± 15 U/L) (Figure S3). These shifts suggest a possible mild hepatic response without broad systemic effects following IN exposure [53, 54]. In the IV groups, clearer alterations were observed (Figures S4 and S5). Both WBC (25 nm: (3 ± 1) × 10^3^/µL, *p* = 0.05, 250 nm: (2 ± 1) × 10^3^/µL, *p* = 0.02, 1000 nm: (2.2 ± 0.5) × 103/µL, *p* = 0.01, control: (4.3 ± 0.9) × 10^3^/µL) and PLT counts (25 nm: (408 ± 175) × 10^3^/µL, *p* = 0.02, 250 nm: (302 ± 106) × 10^3^/µL, *p* = 0.001, 1000 nm: (411 ± 140) × 10^3^/µL, *p* = 0.008, control: (679 ± 108) × 10^3^/µL) were lower in the nanoparticle‐exposed mice compared with controls, whereas RBC and HCT remained within the normal range. In the serum biochemistry panel, most parameters remained within normal ranges, similar to the IN groups, although AST was modestly but significantly elevated in the 25 and 250 nm groups (25 nm: 348 ± 97 U/L, *p* = 0.008, 250 nm: 325 ± 69 U/L, *p* = 0.005, control: 153 ± 57 U/L). ALT showed a non‐significant increase in the 250 nm group (95 ± 68 U/L, *p* = 0.1 vs 36 ± 28 U/L in controls), reflecting a similar pattern of variation observed for IN exposure [53, 54]. Additionally, serum glucose level was slightly reduced relative to controls (25 nm: 125 ± 27 mg/dL, *p* = 0.03, 250 nm: 143 ± 32 mg/dL, *p* = 0.06, 1000 nm: 166 ± 31 mg/dL, *p* = 0.2, control: 209 ± 46 mg/dL), especially noticeable in the 25 nm group, suggesting a route‐ and size‐responsive metabolic effect that was not apparent after IN dosing [55]. Therefore, repeated IN exposure for one week (3 doses) caused only minor hematological changes and limited alterations in serum biochemistry. IV administration was shown to have a possible effect on circulating WBC and PLT counts, together with notable variations in AST and ALT. These results suggest that airway delivery resulted in minimal systemic effects under these conditions, while the IV route provoked more obvious systemic responses, and that the magnitude of these effects depends on particle size.

### 2.6 Maternal and Fetal Exposure

For both administration routes (IN and IV), the accumulation of [^14^C]PS NPs in the placentae and fetuses was low. Within this low range, no consistent size dependence was evident for fetal values, and placental signals did not follow a uniform size‐dependent pattern. These findings signify limited transplacental transfusion under the present exposure conditions.

In late gestation (GD17), tight paracellular junctions and selective vesicular trafficking in the placenta constrain NP transfer. Route‐specific disposition further lowers the maternal driving concentration at the fetoplacental barrier: intranasal dosing is lung‐first with pronounced airway‐to‐GI transit, whereas intravenous dosing is RES‐first with rapid hepatic sequestration (Figures 3C and 4C), yielding low circulating activity at 24 h (Figure 4B).

Two factors frame the interpretation: pooled developmental matrices at the litter level (compressing inter‐fetal variance) and the absence of perfusion (rendering placental measures upper‐bound estimates given intravascular retention). Under these conditions, placental uptake was low and fetal values were below the LOD, reinforcing the conclusion of restricted fetal exposure.

Overall, the developmental data support a barrier‐limited scenario in late gestation: despite route‐specific maternal distributions, neither route yielded considerable fetal uptake for pristine PS NPs of the 25–1000 nm size range. Even in the “worst‐case” scenario (single high dose intravenous administration of [^14^C]PS NPs), placental and fetal signals remained low at 24 h. However, one time dose is not an exact recapitulation of a real‐world scenario where humans are exposed to nanoplastics daily. Environmental exposures to micro‐/nanoplastics are expected to be chronic and typically occur at substantially lower daily masses than the detection‐enabled doses used here. Accordingly, the IN and IV dose levels were selected to enable quantitative tissue‐level mass balance by LSC at 24 h post‐administration – particularly in low‐burden matrices such as placenta and fetal tissues – and to provide route‐ and size‐dependent bounds on biodistribution and placental transfer rather than to replicate environmental concentration profiles. Oral exposure is a major environmental route for micro‐/nanoplastics; however, the present work focused on IN and IV delivery to provide complementary bounds on (i) respiratory deposition with airway‐to‐GI transit and (ii) systemic circulation–driven placental transfer. Future studies should apply this radiotracing platform to oral and chronic low‐dose exposure paradigms to better bridge controlled biokinetic measurements with real‐world exposure scenarios. Consistent with this goal, the analytical sensitivity of the LSC workflow (LOD = 123 DPM/55 pCi, mean blank ± 3 SD) imposes practical constraints on quantifying placental/fetal burdens under environmentally realistic low‐dose regimens at short timepoints. Future work should extend this platform to lower‐dose, longer‐duration exposures and additional environmentally relevant routes to better bridge controlled biokinetic measurements with real‐world exposure scenarios. Even if only a small fraction ends up in our blood flow daily and from there an even smaller fraction accumulates in the placenta, it is still plausible that some plastic particles will end up in the human placenta during pregnancy. This horrifying speculation was recently confirmed by Ragusa et al., who reported that human placental samples contained microplastic particles [17].

While placental and fetal burdens were low under the conditions tested here, prior studies indicate that maternal exposure to PS nanoplastics can still be associated with measurable offspring outcomes, including organ‐specific injury. For example, maternal PS‐NP exposure during gestation/lactation has been linked to hepatic toxicity in male offspring, and gestational PS‐NP exposure has been reported to induce sex‐specific small intestinal toxicity in offspring [56, 57]. These reports help contextualize the present biodistribution findings by underscoring that even limited fetal exposure, if it occurs repeatedly over pregnancy, may still be biologically relevant.

## 3 Conclusions

Here, we have tracked the biodistribution of ^14^C‐labeled polystyrene nanoparticles in late‐gestation mice after intranasal and intravenous exposures. Overall, our data demonstrates that the route of exposure, along with the clearance barriers encountered initially, predominantly affects the NP biodistribution. After intranasal administration, PS NPs exhibited pulmonary retention and excretion through the GI tract, whereas after intravenous injection, accumulation in the liver, spleen, and lungs was observed. We did not detect ^14^C in placentae or fetuses after intranasal administration. After intravenous administration, around 0.10%–0.15% of the injected polystyrene particles were detected in the placentae with all particle sizes. Our results indicate that a small amount of polystyrene particles in the blood flow may accumulate in the placenta. However, the portion of plastic particles in the blood flow may remain low, consistent with rapid tissue association and/or clearance processes reported for nanoparticles in mammalian systems. The radiolabeling method used here – covalent incorporation of ^14^C – enables the quantification of PS NP biodistribution without altering the intrinsic physico‐chemical properties of the PS particles. This radiolabeling technique could be extended to other polymer materials as well, and more importantly, be used in chronic exposure investigations that are required to fully recapitulate the real‐world human exposure.

## 4 Experimental Section

### 4.1 Chemicals and Materials

Styrene (99.5%, stabilized, for analysis), ammonium persulfate (APS), sodium hydroxide (NaOH), and ethanol were purchased from ThermoScientific (Waltham, MA, USA) and used without further purification. Potassium persulfate (KPS, ThermoScientific, Waltham, MA, USA) was recrystallized in deionized water prior to use. Phosphate‐buffered saline (PBS, 10X solution, ThermoScientific, Waltham, MA, USA) was diluted ten‐fold prior to use. Sodium dodecyl sulfate (SDS, MP Biomedicals, Solon, OH, USA) was used as received. Polyvinylpyrrolidone (PVP, Mw = 40,000 g mol^−1^) was obtained from TCI Chemicals (Tokyo, Japan). Radiolabeled styrene ([methylene‐[^14^C]styrene in methylene chloride, specific activity 2.035 GBq mmol^−^^1^, Mw = 106.14 g mol^−1^) was obtained from American Radiolabeled Chemicals Inc. (St. Louis, MO, USA) and used as received. Soluene‐350 and Hionic‐Fluor were purchased from Revvity (Waltham, MA, USA). All aqueous solutions were prepared using Milli‐Q ultrapure water (resistivity ≥18.2 MΩ·cm, Millipore, Burlington, MA, USA).

### 4.2 Preparation of [^14^C]Polystyrene Nanoparticles of Different Sizes

PS NPs with nominal diameters of 25, 250, and 1000 nm were prepared via free‐radical polymerization of styrene in aqueous media under a nitrogen atmosphere, following previously established protocols with minor modifications for nanoscale synthesis.

The ≈25 and ≈250 nm particles were produced according to the method described previously [58]. Radiolabeled [^14^C]PS NP of 25 nm were synthesized by conventional emulsion polymerization with 200 µg total styrene (100 µg styrene + 100 µg [methylene‐[^14^C]styrene in 100 µL methylene chloride, final activity was 370 kBq), SDS 200 mg in 9.7 mL water, and KPS 9.44 mg (3.60 mm in the aqueous phase), the mixture was N_2_ purged for 30 s and hold at 75°C. For 250 nm [^14^C]PS NPs – surfactant‐free emulsion with 200 µg total styrene (100 µg styrene + 100 µg [methylene‐[^14^C]styrene in 100 µL methylene chloride, final activity was 370 kBq) in 6.08 g water, initiator solution KPS 31 mg + NaOH 17.8 mg in 1.786 g water (final water 7.866 g, 14.6 mM KPS, 56.7 mm NaOH), the mixture was N_2_ purged for 30 s, heated to 75°C, and after initiator injection, hold overnight. The ≈1000 nm particles were synthesized using a modified dispersion polymerization approach [59], where 400 µg total styrene (200 µg styrene + 200 µg [methylene‐[^14^C]styrene in 200 µL methylene chloride, final activity was 740 kBq) was added to 2.5 mL ethanol containing 1 mg PVP, N_2_‐purged 30 s, and heated to 70°C. APS (4 mg in 0.30 mL) water was then injected, and the reaction was maintained at 70°C for 18 h.

After polymerization, dispersions were cooled to room temperature and dialyzed against Milli‐Q water using Float‐A‐Lyzer G2 devices (20 kDa MWCO, Repligen, Rancho Dominguez, CA, USA). The resulting [^14^C]PS stocks had a specific activity of 100 nCi mg^−^^1^, and final NP concentrations were determined from ^14^C activity by LSC (Hidex 300 SL, Turku, Finland).

### 4.3 [^14^C]Polystyrene Nanoparticle Characterization

Hydrodynamic diameters were determined by dynamic light scattering (DLS) in water at 25°C on a Zetasizer Nano ZS (Malvern Panalytical, Westborough, MA, USA) using backscatter detection at 173°, the z‐average (intensity‐weighted) hydrodynamic diameter and polydispersity index (PDI) are reported (*n* = 3 per batch). ζ‐potential was measured by electrophoretic light scattering (25°C, disposable folded capillary cell, 173°, Smoluchowski model, *n* = 3 per batch) in deionized water. Transmission electron microscopy (TEM) images are provided in Figure S1 to document particle morphology and dispersion quality. Due to shared facility radiation safety restrictions, electron microscopy was performed on non‐radioactive (“cold”) PS nanoparticles synthesized using the identical protocol and purification workflow as the [^14^C]PS batches; the ^14^C label is incorporated at trace levels in the polymer backbone and does not introduce surface functionalization. Hydrodynamic diameter and dispersity (DLS/PDI/ ζ‐potential) were measured for both nonradioactive (Figure S1) and radioactive particles to confirm similar size and dispersion of the materials.

To assess radiochemical/chemical stability, NPs were incubated at 37°C for 14 days in PBS, simulated lung fluid (SL), simulated gastric fluid (SGL), simulated intestinal fluid (SIL), and mouse serum. Aliquots were analyzed by instant thin‐layer chromatography on silica‐gel–impregnated glass microfiber paper (iTLC‐SG, Agilent Technologies, Folsom, CA, USA) developed with n‐hexane/ethyl acetate (95:5, v/v) and quantified by LSC. Under this solvent system, styrene migrated to the solvent front (R_f ≈0.9–1.0), whereas [^14^C]PS remained at the origin. The limit of detection (LOD) for [^14^C]styrene was defined as mean blank + 3 × standard deviation (SD).

### 4.4 Animals

Timed‐pregnant C57BL/6J mice (The Jackson Laboratory, Bar Harbor, ME, USA) arrived on gestational day 11 (GD11). Mice were randomly assigned to a treatment group upon arrival. On the day of necropsy, dams weighed 32 ± 2 g (mean ± SD). Mice were group‐housed (≤5 per cage) in individually ventilated cages with standard chow and water ad libitum, on a 12:12 h light–dark cycle at 22 ± 1°C. Euthanasia was performed under deep isoflurane anesthesia in accordance with AVMA Guidelines and UAB IACUC policy. All procedures were approved by the University of Alabama at Birmingham IACUC (Animal Welfare Assurance Number: D16‐00162, protocol number: IACUC‐23113) and conducted in accordance with relevant guidelines and regulations, reporting follows ARRIVE.

### 4.5 Dosing and Administration

Two exposure routes were evaluated with PBS as a control. For intranasal (IN) exposure, dams were randomly assigned to receive [^14^C]PS NPs at nominal diameters of 25, 250, or 1000 nm (*n* = 6 per size) or PBS control (*n* = 4). Dosing occurred on GD12, GD14, and GD16 (three doses total). Each IN dose consisted of 20 µL containing 0.5 mg of [^14^C]PS and 50 nCi of activity. Dams were euthanized 24 h after the final IN dose (GD17), followed by necropsy and sample collection.

The intranasal exposure schedule (GD12, GD14, and GD16) was selected to provide multiple exposures during late gestation, a period of rapid fetal growth and mature placental function, while remaining feasible and safe for pregnant dams. Exposures were spaced every 48 h to achieve a repeated‐dose regimen within a short late‐gestation window and to limit the frequency of anesthesia and handling associated with intranasal instillation, which can increase stress and procedural risk during pregnancy. Thus, this regimen represents a controlled late‐gestation repeated‐exposure design rather than a direct simulation of continuous daily real‐world exposure.

For intravenous injection, a separate set of dams was randomly assigned to receive [^14^C]PS NPs at 25, 250, or 1000 nm (*n* = 6) or PBS control (*n* = 4). A single bolus was administered on GD16, the injection volume was 100 µL, and it delivered 1.5 mg of [^14^C]PS and 150 nCi of activity per dam. Dams were euthanized 24 h post‐injection (GD17), followed by necropsy and sample collection.

Stock suspensions of synthesized [^14^C]PS nanoparticles were stored in a temperature‐controlled environment at 2–8°C until use. To minimize time‐dependent changes in dispersion state, working dilutions in sterile PBS were prepared immediately before each administration. Before dosing, suspensions were mixed by vortexing and briefly sonicated to promote uniform dispersion and reduce agglomeration, suspensions were then administered promptly after preparation. No visible precipitation was observed during the dosing period; however, as with many colloids in electrolyte solutions, some degree of time‐dependent aggregation in PBS cannot be fully excluded, which is why dilutions were prepared fresh and dispersed immediately before administration.

Dose levels and schedules were selected to enable quantitative tissue‐level mass balance by liquid scintillation counting (LSC) in both high‐burden and low‐burden matrices (including placenta and fetal tissues) at 24 h post‐administration, while maintaining a consistent tracer specific activity across conditions. Although precise human exposure levels remain uncertain and method‐dependent [60, 61, 62, 63, 64], mass‐based estimates of human microplastic intake have been reported on the order of ≈0.1–5 g/week [63]. Under simple body‐weight normalization, this range corresponds to ≈0.03–1.4 mg/week for a 20 g mouse, providing order‐of‐magnitude context for the doses used here. Accordingly, the IN regimen (0.5 mg on GD12, GD14, and GD16, total 1.5 mg over one week) was designed to represent repeated respiratory deposition during a developmentally sensitive window, whereas the IV bolus (1.5 mg on GD16) was included as a systemic “upper‐bound” condition to constrain placental transfer following direct access to the maternal circulation.

### 4.6 Ex Vivo Biodistribution and ^14^C Radioactivity Measurement

Immediately after euthanasia, whole blood was collected by cardiac puncture, and dams were dissected. The following biological matrices were collected:

1. Organs/tissues: heart, lungs, liver, gallbladder, stomach, pancreas, spleen, salivary gland, small and large intestines (with luminal contents), kidneys, skeletal muscle, brain, spinal cord, and ovaries.
2. Fluids: blood.
3. Excreta: feces.
4. Developmental samples: whole fetuses (GD 17) and placentae.

All matrices from NP exposed and PBS control cohorts were weighed and completely solubilized in Soluene‐350 at 40°C for 1–3 days (duration scaled to sample mass). Following digestion, aliquots were processed as described in Table S1 to ensure compatibility with LSC. Developmental matrices (fetuses, placentae) were pooled per litter, two dams had no recoverable fetuses/placentae at necropsy (developmental *n* = 39 litters). Then, all samples were mixed with 18 mL of Hionic‐Fluor scintillation cocktail in 20 mL low‐potassium glass vials and dark‐adapted for 72 h to allow residual H_2_O_2_‐induced chemiluminescence to decay and stabilize counts.

^14^C activity was quantified by LSC using a Hidex 300 SL (Hidex, Turku, Finland) operating in triple‐PMT TDCR mode with chemiluminescence rejection (absolute efficiency, no external standards). Counting parameters were: 600 s per vial, coincidence resolving time 35 ns, Ionized Delay 2 s, Chamber Delay 5 s, and the ^14^C β‐window set to ROI1 channels 5–650 (one acquisition per vial). Quench indices were recorded for each vial. Background was determined from Hionic‐Fluor cocktail blanks (mean 90 DPM, 41 pCi) and subtracted from all samples; reported values are background‐corrected. The detection limit was 123 DPM (55 pCi), defined as the mean blank signal plus three standard deviations. This sensitivity informed dose selection to ensure measurable signals in low‐burden tissues.

Percent of injected/instilled dose (%ID) and mass‐normalized %ID per gram (%ID/g) were computed as:

$$\%ID=\frac{A_{sample}}{ID_{total}}\;\;\times \;100\%$$

$$\%\;\frac{ID}{g}=\frac{A_{sample}}{ID_{total}\;\times \;mass}\;\;\times \;100\%$$

where *A_sample_* is the activity measured for the sample, *ID_total_* is the total administered activity per dam, and *mass* is the matrix mass (g). For developmental matrices (placentae, fetuses), *A_sample_* and *mass* are reported per litter.

### 4.7 Blood Analysis

Terminal whole blood was collected by cardiac puncture under deep isoflurane anesthesia immediately after euthanasia. For clinical chemistry, blood was allowed to clot for 30 min at room temperature and centrifuged at 1,000 g for 10 min; serum was analyzed the same day on a Heska Element DC5X (Heska, Loveland, CO, USA). Analytes included blood urea nitrogen (BUN), creatinine, BUN/creatinine ratio, phosphorus, calcium, total protein, albumin, globulin, albumin/globulin ratio, glucose, alanine aminotransferase (ALT/GPT), aspartate aminotransferase (AST/GOT), alkaline phosphatase (ALP), and total bilirubin.

For hematology, whole blood was collected into Microvette 100 K3E microtubes (K_3_‐EDTA, Sarstedt Inc., Newton, NC, USA) and analyzed on a Heska Element HT5+ (Heska, Loveland, CO, USA). Reported parameters: white blood cell count (WBC), red blood cell count (RBC), hematocrit (HCT), and platelets (PLT). Analyses used manufacturer mouse settings; results are reported in standard clinical units. Quality control followed manufacturer recommendations (two‐level controls per run, routine calibration/maintenance), samples with hemolysis/lipemia or instrument flags were re‐run, diluted, or excluded per predefined criteria.

### 4.8 Statistical Analysis

Statistical analyses were performed in GraphPad Prism (10.4.2). For each exposure route and organ (*n* = 6), planned pairwise comparisons among particle sizes (25 vs 250 nm, 25 vs 1000 nm, and 250 vs 1000 nm) were conducted using unpaired two‐tailed Welch's t‐tests. Given the number of tissue endpoints evaluated, *p*‐values were adjusted for multiple comparisons using the Holm–Šídák method. Adjusted *p*‐values < 0.05 were considered statistically significant. Corrections were applied separately for %ID and %ID/g endpoints within each exposure route. For hematology and serum biochemistry (*n* = 4–6), each parameter in particle‐exposed groups was compared with PBS controls using unpaired two‐tailed tests (Welch's correction). A two‐sided *p*‐value < 0.05 was considered statistically significant. Data is presented as mean ± SD.

## Funding

The authors gratefully acknowledge the National Institutes of Health (R00ES034053).

## Conflicts of Interest

The authors declare no conflict of interest.

## Supporting information




**Supporting File**: advs74793‐sup‐0001‐SuppMat.pdf.

## Data Availability

The data that support the findings of this study are available in the supplementary material of this article.
