# Supplementary material for: Tracking of [14C]Polystyrene Nanoplastics in Pregnant Mice
Source: Adv Sci (Weinh). 2026 Mar 24;13(32):e23995. doi: 10.1002/advs.202523995 (PMC13252641; doi:10.1002/advs.202523995)
Supplement: Supplementary file 1 — Supporting File: advs74793‐sup‐0001‐SuppMat.pdf. [file ADVS-13-e23995-s001.pdf]

Supporting Information

Tracking of [^{14}C]polystyrene nanoplastics in pregnant mice

Olga Khaybullina[1], Pallavi Sarkhel[1], and Outi M. Keinänen[1,2,]*

[1]Department of Chemistry, University of Alabama at Birmingham, Birmingham, AL, 35294, USA

[2]Department of Radiology, University of Alabama at Birmingham, Birmingham, AL, 35294, USA

***Corresponding Author**: Outi Keinänen; 901 14th Street South, Birmingham, AL, 35294, USA; Phone: 205-9759410; E-mail: keinanen@uab.edu

Table S1. Preparation of *ex vivo* biological matrices for ^{14}C quantification by LSC.

Matrix[a-c]	Typical mass (g), mean ± SD	Soluene-350 (mL per sample)	Soluene-to-mass (mL/g)	Digestion (days)	Aliquot to vial (mL)	H_2O_2 added (mL; 30% w/w)
Blood (whole)[e]	0.01 ± 0.008	2	195.2	1	1	0.1
Brain	0.335 ± 0.102	2	6	2	1	0.2
Feces[e]	0.032 ± 0.016	2	63.2	2	1	0.1
Fetuses (GD17)[e]	0.925 ± 1.27	4	4.3	3	1	0.2
Gallbladder[e]	0.008 ± 0.007	1	120.6	1	1	0[g]
Heart	0.123 ± 0.036	2	16.3	2	1	0.2
Kidneys	0.32 ± 0.086	5	15.6	3	1	0.1
Large intestine[d]	0.73 ± 0.21	5	6.9	3	1	0.2
Liver	1.652 ± 0.448	10	6.1	3	1	0.2
Lungs	0.194 ± 0.061	2	10.3	2	1	0.1
Muscle	0.081 ± 0.041	2	24.6	1	1	0.1
Ovaries	0.125 ± 0.056	2	16	2	1	0.2
Pancreas	0.214 ± 0.091	2	9.4	2	1	0.1
Placentae (GD17)[f]	1.435 ± 0.535	5	3.5	3	1	0.3
Salivary gland	0.061 ± 0.029	1	16.5	1	1	0.1
Small intestine[f]	1.187 ± 0.341	5	4.2	3	1	0.2
Spinal cord	0.039 ± 0.021	1	25.8	1	1	0.1
Spleen	0.106 ± 0.031	2	18.8	2	1	0.2
Stomach	0.35 ± 0.119	5	14.3	3	1	0.2

[a] Maternal matrices processed for LSC: n = 41 dams total (25 nm: 11; 250 nm: 12; 1000 nm: 12; PBS controls: 6). All maternal matrices were available for all dams.

[b] Developmental matrices (fetuses, placentae) were pooled per litter; n = 39 litters (two dams had no recoverable fetuses/placentae at necropsy; miscarriage/early delivery suspected).

[c] Values are wet mass post-perfusion; mean ± SD across all animals (exposed and PBS controls).

[d] Intestinal masses include luminal contents; feces are wet mass.

[e] For small-mass matrices (e.g., blood, gallbladder, feces), Soluene-to-mass ratios appear large because a minimum Soluene volume is required for complete solubilization irrespective of mass.

[f] Fetuses and placentae are from gestational day 17 (GD 17); pooling/individual analysis is described in 2.6.

[g] H_2O_2 was omitted for gallbladder samples.

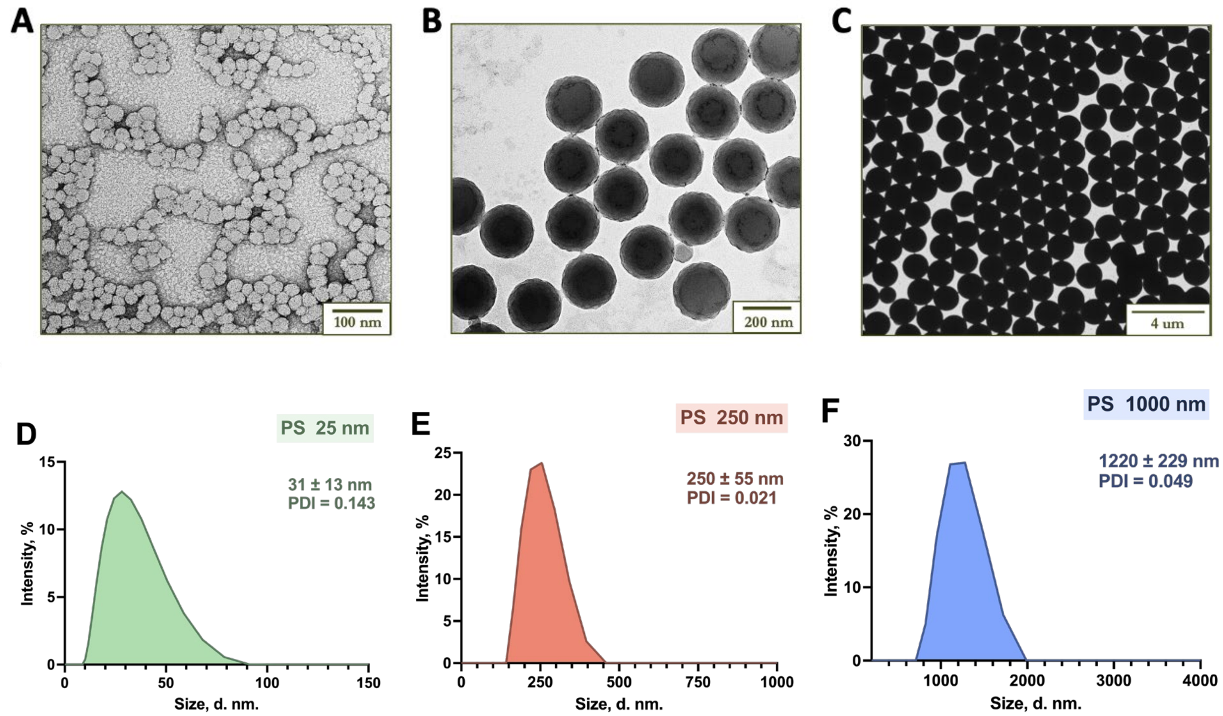

Figure S1. Characterization of synthesis-matched non-radioactive ("cold") PS nanoparticles corresponding to the [^{14}C]PS materials. Representative TEM micrographs of cold PS nanoparticles prepared using the same emulsion polymerization and purification protocol as the radiolabeled [^{14}C]PS used in this study. Images confirm spherical morphology and dispersion quality for each nominal size. Direct TEM imaging of radiolabeled materials was not permitted under shared facility radiation-safety policies; therefore, TEM of synthesis-matched cold particles is provided as a morphology reference for particles produced under identical conditions. (**A–C**) TEM images of PS nanoparticles with nominal diameters of 25 nm (**A**), 250 nm (**B**), and 1000 nm (**C**). (**D–F**) Corresponding DLS intensity-weighted size distributions with mean hydrodynamic diameter (mean ± SD) and PDI: 25 nm (**D**), 31 ± 13 nm; PDI = 0.143; 250 nm (**E**), 250 ± 55 nm; PDI = 0.021; 1000 nm (**F**), 1220 ± 229 nm; PDI = 0.049. Zeta potentials (mean ± SD) were −36 ± 5 mV (25 nm), −35 ± 4 mV (250 nm), and −41 ± 8 mV (1000 nm).

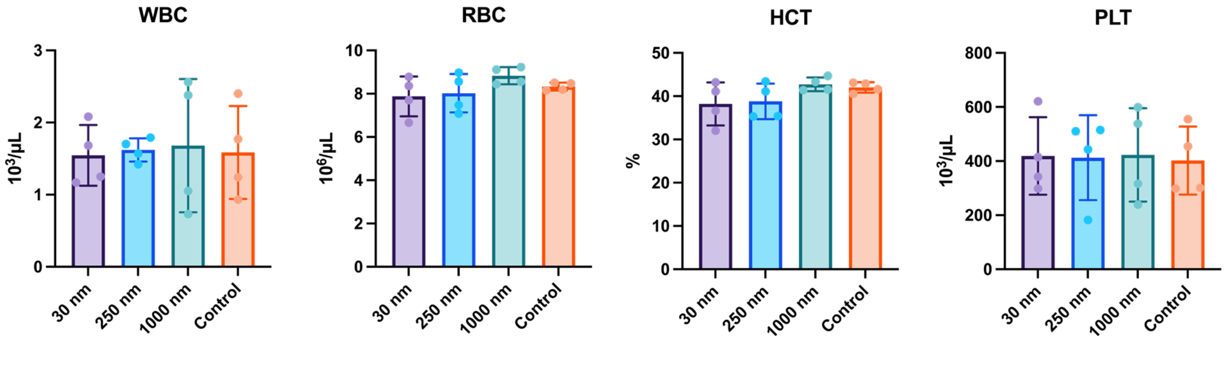

Figure S2. Hematological parameters following intranasal (IN) exposure. White blood cell (WBC), red blood cell (RBC), hematocrit (HCT), and platelet (PLT) counts in dams 24 h after repeated IN administration of polystyrene nanoparticles (3 doses over one week). Values are presented as mean ± SD.

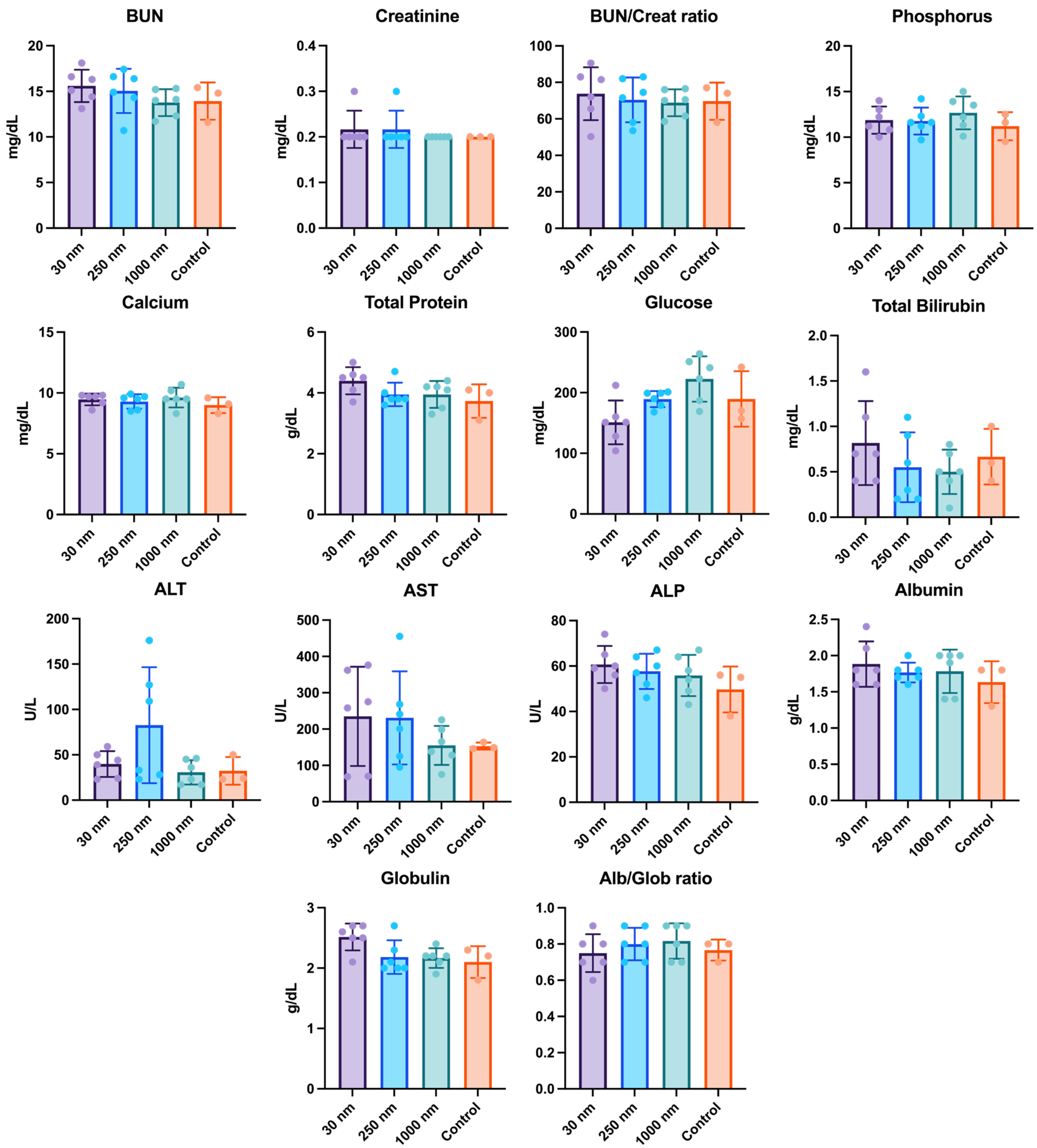

Figure S3. Serum chemistry markers following intranasal (IN) exposure. Blood urea nitrogen (BUN), creatinine, BUN/creatinine ratio, phosphorus, calcium, total protein, albumin, globulin, albumin/globulin ratio, glucose, alanine aminotransferase (ALT/GPT), aspartate aminotransferase (AST/GOT), alkaline phosphatase (ALP), and total bilirubin measured 24 h after repeated IN administration of polystyrene nanoparticles (3 doses over one week). Values are presented as mean ± SD.

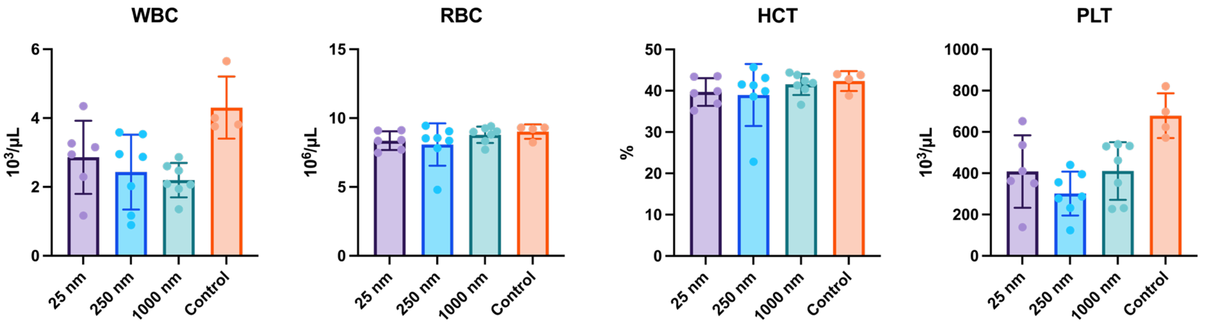

Figure S4. Hematological parameters following intravenous (IV) exposure. White blood cell (WBC), red blood cell (RBC), hematocrit (HCT), and platelet (PLT) counts in dams 24 h after a single IV injection of polystyrene nanoparticles. Values are presented as mean ± SD.

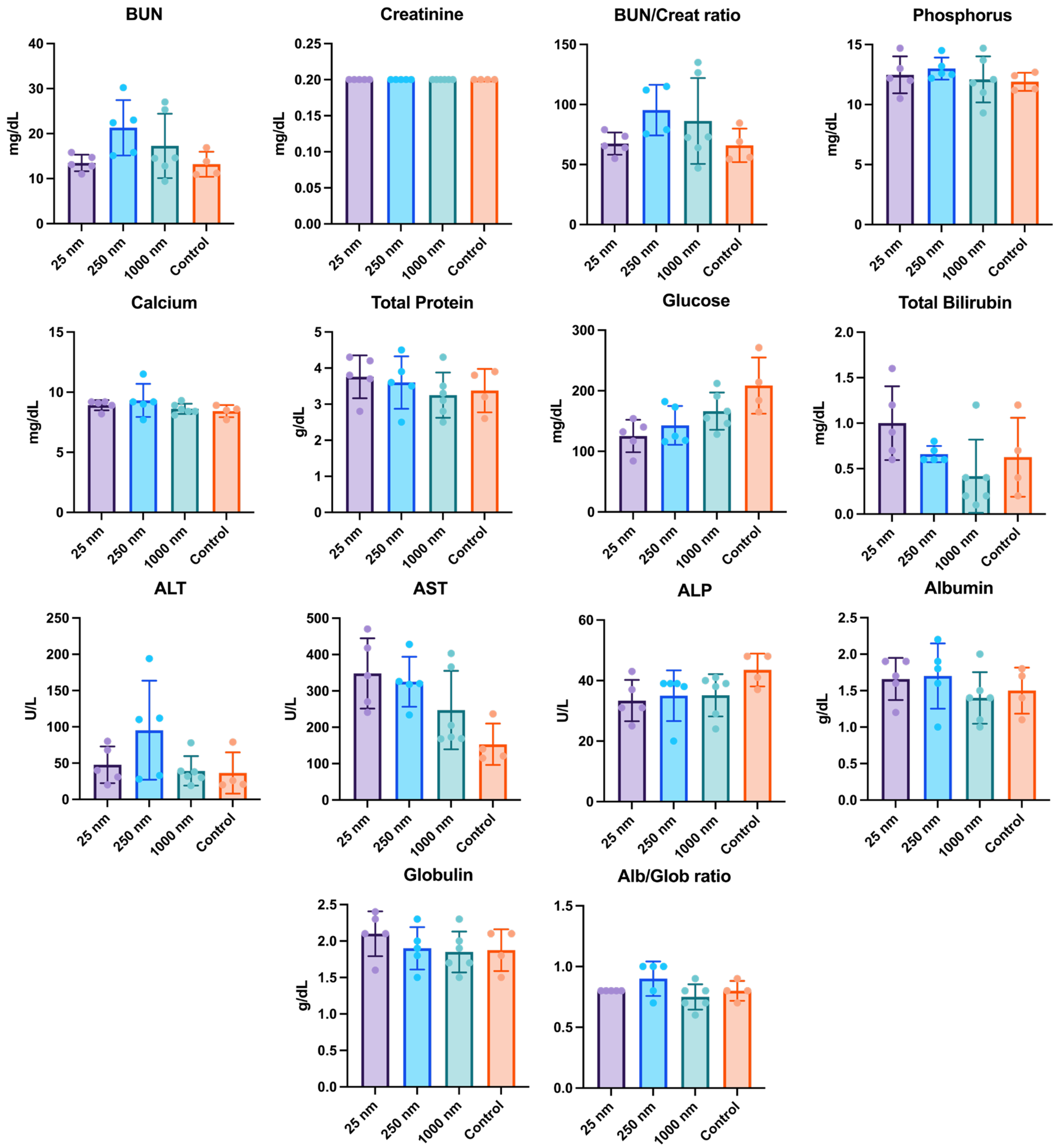

Figure S5. Serum chemistry markers following intravenous (IV) exposure. Blood urea nitrogen (BUN), creatinine, BUN/creatinine ratio, phosphorus, calcium, total protein, albumin, globulin, albumin/globulin ratio, glucose, alanine aminotransferase (ALT/GPT), aspartate aminotransferase (AST/GOT), alkaline phosphatase (ALP), and total bilirubin measured 24 h after IV injection of polystyrene nanoparticles. Values are presented as mean ± SD.
